# Improved incidence estimates from linked vs. stand-alone electronic health records

**DOI:** 10.1016/j.jclinepi.2016.01.005

**Published:** 2016-07

**Authors:** Elizabeth R.C. Millett, Jennifer K. Quint, Bianca L. De Stavola, Liam Smeeth, Sara L. Thomas

**Affiliations:** a Department of Infectious Disease Epidemiology, Faculty of Epidemiology and Population Health, London School of Hygiene and Tropical Medicine, Keppel Street, London WC1E 7HT, UK; b Department of Non-communicable Disease Epidemiology, Faculty of Epidemiology and Population Health, London School of Hygiene and Tropical Medicine, Keppel Street, London WC1E 7HT, UK; c Department of Medical Statistics, Faculty of Epidemiology and Population Health, London School of Hygiene and Tropical Medicine, Keppel Street, London, WC1E 7HT, UK

**Keywords:** Pneumonia, Electronic health records, Data linkage, Aged, England/epidemiology, Cohort

## Abstract

**Objective:**

Electronic health records are widely used for public health research, and linked data sources are increasingly available. The added value of using linked records over stand-alone data has not been quantified for common conditions such as community-acquired pneumonia (CAP).

**Study Design and Setting:**

Our cohort comprised English patients aged ≥65 years from the Clinical Practice Research Datalink, eligible for record linkage to Hospital Episode Statistics. Stand-alone general practice (GP) records were used to calculate CAP incidence over time using population-averaged Poisson regression. Incidence was then recalculated for the same patients using their linked GP-hospital admission data. Results of the two analyses were compared.

**Results:**

Over 900,000 patients were included in each analysis. Population-averaged CAP incidence was 39% higher using the linked data than stand-alone data. This difference grew over time from 7% in 1997 to 83% by 2010. An increasingly larger number of pneumonia events were recorded in the hospital admission data compared to the GP data over time.

**Conclusion:**

Use of primary or secondary care data in isolation may not give accurate incidence estimates for important infections in older populations. Further work is needed to establish the extent of this finding in other diseases, age groups, and populations.

**What is new?**

**Key findings**
• Use of linked primary-secondary care health data provided markedly higher incidence estimates of community-acquired pneumonia compared to stand-alone general practice (GP) records for the same group of English older adults.
• Comparison of the data sources revealed diverging incidence estimates over time, rising from 7% higher in 1997/98 to 83% higher in 2010/11 when using the linked data compared to the stand-alone GP data.

**What this adds to what was known?**
• The benefits of the use of linked electronic health records (compared to single data sources) have been demonstrated for conditions such as cardiovascular diseases; this is the first article to demonstrate the benefits for an important, common infection.

**What is the implication and what should change now?**
• Use of primary or secondary care data in isolation may not give accurate estimates of burden of disease for important infections in older populations.
• Further work is needed to establish if this trend is seen in other infections and diseases.

## 1 Introduction

Electronic health records are extensively used in epidemiological research, because of their wide and detailed population coverage. It is increasingly possible to link electronic data sources to enhance available data. For example, linked primary and secondary care data provide more complete information on outcomes, enriched data on covariates such as patients' medical and therapeutic histories, and accurate timing of events such as hospitalizations. The value of linked over stand-alone data has been investigated for conditions such as cardiovascular events, asthma, diabetes, and upper gastrointestinal bleeding [1], [2], [3], [4]. However, the potential benefits of linked data for examining the burden of important infectious diseases are unclear.

Community-acquired pneumonia (CAP) causes considerable morbidity among older individuals and can be treated in either primary or secondary care. Large-scale studies of CAP incidence trends have commonly used either stand-alone general practice (GP) records, potentially excluding patients who present to hospital if practices record hospitalized events suboptimally, or stand-alone hospital records which exclude cases treated in the community. Two recent studies used large linked GP and hospital data sets to assess disease burden of CAP but did not assess the added value of using the linked data [5], [6].

We thus investigated the utility of linked primary/secondary care data in better determining trends in CAP disease burden in England among those aged ≥65 years by comparing incidence of CAP derived from stand-alone primary care data with that from linked primary-secondary care data. Each analysis used essentially the same cohort of patients over the same time period, using the same analytical approach.

## 2 Methods

The Clinical Practice Research Datalink (CPRD) is a nationally representative UK primary care dataset, containing a range of information including Read-coded diagnoses [1]. Hospital Episode Statistics (HES) contain inpatient records with ICD10-coded diagnoses, including admission and discharge dates. CPRD and HES records are linked at a patient-level for consenting English practices. By March 2011, CPRD contained >12 million patient records, with HES-linkage available for 65% of English CPRD practices (around 5% of the English population) [7].

Practices and patients joined CPRD throughout the study period, providing dynamic cohorts of patients. To ensure comparability of the two data sources, a near-identical group of patients were used in both analyses. Patients included in the study were eligible for record linkage, were aged ≥65 years, and contributed ≥1 day of follow-up. Follow-up started at the latest of the study start date (April 1, 1997), the patient's 65th birthday, the date the practice met CPRD quality standards or 28 weeks after patient registration (to exclude historical illnesses retrospectively reported) [6]. Follow-up ended at the earliest of the study end date (March 31, 2011), death, the practice's last data collection date, or the date the patient left the practice.

We have previously described in detail definitions for pneumonia illness episodes in CPRD and HES, using pneumonia and other lower respiratory tract infection records [6]. In brief, records for which pneumonia was recorded in CPRD (stand-alone and linked data) or as the admitting diagnosis (primary code of the first episode) in HES (linked data only) within 28 days of each other or of a record for lower respiratory tract infection were considered to be part of the same episode. The incident date of the episode was the date of the first of these pneumonia codes.

In both analyses, pneumonia illness episodes which started ≤14 days after a hospitalization were assumed to be hospital-acquired (HAP) and were excluded; episodes with no such hospitalization record were classed as community acquired. The method for defining hospitalizations, and thus distinguishing between CAP and HAP, differed between the two analyses. In the stand-alone CPRD data, hospitalization records were identified using Read codes and other relevant fields in the GP files. In the linked cohort, the 14-day period started at the discharge date of any hospital admission.

Patients were not considered “at-risk” of pneumonia during any pneumonia episode (CAP or HAP) or for 28 days after the last record in the episode, and this time was excluded from the denominator in both cohorts. A key difference in the linked data analysis was the capacity to also exclude the duration of any hospital admission and the subsequent 14 days from person-time at risk of a community-acquired infection and thus obtain more accurate denominator data. This was not possible in the stand-alone data as hospital admission, and discharge dates were not available.

Population-averaged Poisson models were used to calculate the incidence of CAP across clusters of CAP episodes per patient. Rates were calculated stratified by year, age group, and sex.

The financial year structure (April 1–March 31) was used to assign respiratory pathogens circulating over winter months to the same year.

In the linked data, whether patients had consulted with a GP (either face to face or by telephone) on the CAP incident date was examined using the “constype” field in the consultation file.

## 3 Results

The study population included 917,852 patients in the stand-alone data from 351 practices across England. The linked analysis included 916,128 (>99.8%) of these patients who had ≥1 day of follow-up after additionally excluding person-time in hospital. In both analyses 53% of patients were aged 65–69 years at start of follow-up and 56% were female. Using only GP records, we identified 31,575 CAP episodes during the study period. Using linked GP/hospital admission data identified 45,285 CAP episodes. In both analyses, >80% of patients had only one CAP episode during follow-up.

**Fig. 1:**
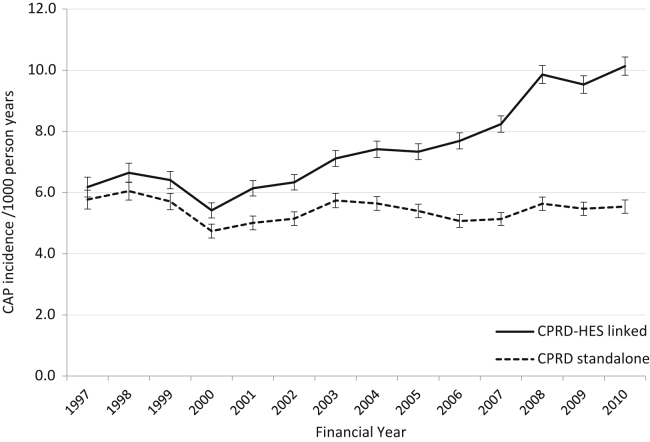
Population-averaged incidence of CAP among older adults by data source over time. *Abbreviations:* CAP, community-acquired pneumonia; CPRD, Clinical Practice Research Datalink; HES, hospital episode statistics.

Incidence estimates using linked data were higher than those using stand-alone data. Overall, incidence was 39% higher using the linked data, and the difference increased markedly over time from 7% (6.18 vs. 5.77/1,000 person-years) in 1997/98 to 83% higher (10.13 vs. 5.54/1,000 person-years) in 2010/11 (Fig. 1). Although rates of CAP rose with age in both data sources, the relative increase in CAP estimates using the linked compared to GP stand-alone data was comparable for each age group, and so, the disparity was not attributable to a specific age group (data not shown). Incidence was higher in men than women in both analyses, but the divergence between estimates was observed in both sexes.

Because of the dynamic nature of the cohort, the number of patients contributing to each analysis increased over the study period, increasing the person-time included. However, the increase in person-time within each analysis was similar (91% increase in linked vs. 93% in stand-alone data), whereas the increase in CAP episodes was substantially larger in the linked data (147% vs. 52% in stand-alone).

Between 1997 and 2010, the percentage of patients who had consulted with their GP on the day of the CAP diagnosis decreased from 82% to 43%. Over the same period, consultation with a GP for an LRTI in the 28 days before the CAP diagnosis decreased from 15% to 10%.

## 4 Discussion

Our investigation of incidence trends for a major infectious disease shows the benefits of using linked data. Use of primary care data alone yielded CAP incidence estimates that were 28% lower than estimates from linked primary/secondary care data. The divergence between estimates increased appreciably over the 14-year study period, and linked data estimates were 83% higher than those from stand-alone GP records by March 2011.

In the linked data analysis, we could refine estimated person-time at risk of community-acquired infection, by discounting the person-time patients were in hospital. However, it seems that the diverging estimates were attributable largely to the higher number of CAP episodes in the linked data. All pneumonias recorded in GP records are included in linked GP/hospital data, but pneumonias from hospital admissions are only included in stand-alone GP data if patients consulted their GP prehospitalization, or hospital diagnoses were retrospectively recorded by the patients' GP. Our analyses demonstrate that CAP identified in hospital is incompletely recorded by GPs, and this underrecording, coupled with the known increase in CAP hospitalizations in England over the study period, may explain the divergence we report [8]. Patients with CAP may have increasingly presented directly to Accident and Emergency Departments because of changes in GP service provision or perceived severity of illness, and the threshold for admission for these older patients may also have decreased. Both these scenarios are consistent with the larger increase in CAP episodes in the HES records and with decreasing consultations with a GP on the day of a CAP diagnosis. They also highlight that for conditions that can be treated both in the community and in hospital, changes to health services, patient, and clinician behavior could all result in marked underestimation of disease burden if single data sources are used.

Our analyses used large, nationally representative data sets containing ≥900,000 patients [9]. Overall validity of diagnoses in CPRD data has been shown to be high, although few studies have assessed the sensitivity of recording [10]. Over 99.8% of the same patients were included in both analyses, enabling examination of the differences in CAP estimates due to the data source and methodology used. We are unaware of other studies that have assessed the added value of using linked vs. stand-alone data within the same population for estimating the burden of any infectious disease.

The two data sources use different coding systems, and changes to coding practices over time within each source are a further consideration. For example, “tentative” pneumonia codes such as “Influenza or pneumonia” (available in the Read but not ICD10 coding system) were not included in this study. Patients assigned a tentative pneumonia code by their GP and subsequently hospitalized with CAP would have been included in the linked data but not in the stand-alone data. However, to have contributed to the disparity, GPs would have needed to use these tentative diagnoses increasingly over time. Alternatively, if hospital physicians increasingly diagnosed or labeled older patients as having pneumonia, this would contribute to the divergent trends. We have no evidence that this occurred, but a clear understanding of trends in coding practices is essential for interpreting findings from both stand-alone and linked data.

In conclusion, use of primary or secondary care data in isolation may underestimate disease incidence for certain conditions, particularly those that can be treated in either care setting. Additionally, incomplete recording of events in UK stand-alone GP data limits its use in studies of the burden of pneumonia in older adults. Further work is needed to establish if this trend is seen in other diseases and age groups.
